# Perirhinal cortex lesions uncover subsidiary systems in the rat for the detection of novel and familiar objects

**DOI:** 10.1111/j.1460-9568.2011.07755.x

**Published:** 2011-07

**Authors:** Mathieu M Albasser, Eman Amin, Mihaela D Iordanova, Malcolm W Brown, John M Pearce, John P Aggleton

**Affiliations:** 1 School of Psychology, Cardiff University70 Park Place, Cardiff CF10 3AT, Wales, UK; 2 MRC Centre for Synaptic Plasticity, Department of Physiology and Pharmacology, University of Bristol, Medical SchoolBristol, UK

**Keywords:** habituation, hippocampus, learning, perirhinal cortex, recognition memory

## Abstract

The present study compared the impact of perirhinal cortex lesions on tests of object recognition. Object recognition was tested directly by looking at the preferential exploration of novel objects over simultaneously presented familiar objects. Object recognition was also tested indirectly by presenting just novel objects or just familiar objects, and recording exploration levels. Rats with perirhinal cortex lesions were severely impaired at discriminating a novel object from a simultaneously presented familiar object (direct test), yet displayed normal levels of exploration to novel objects presented on their own and showed normal declines in exploration times for familiar objects that were repeatedly presented (indirect tests). This effective reduction in the exploration of familiar objects after perirhinal cortex lesions points to the sparing of some recognition mechanisms. This possibility led us to determine whether rats with perirhinal cortex lesions can overcome their preferential exploration deficits when given multiple object familiarisation trials prior to that same (familiar) object being paired with a novel object. It was found that after multiple familiarisation trials, objects could now successfully be recognised as familiar by rats with perirhinal cortex lesions, both following a 90-min delay (the longest delay tested) and when object recognition was tested in the dark after familiarisation trials in the light. These latter findings reveal: (i) the presumed recruitment of other regions to solve recognition memory problems in the absence of perirhinal cortex tissue; and (ii) that these additional recognition mechanisms require more familiarisation trials than perirhinal-based recognition mechanisms.

## Introduction

In a successful test of spontaneous object recognition an animal spends more time exploring a novel object than a simultaneously presented familiar object (Ennaceur & Delacour, 1988). This outcome is not observed in rats with lesions of the perirhinal cortex, which often spend equal amounts of time exploring both objects (Ennaceur *et al.*, 1996; Aggleton *et al.*, 1997; Norman & Eacott, 2004; Barker *et al.*, 2007; Winters *et al.*, 2008). Various theories can explain the foregoing effect (Wiig & Bilkey, 1994; Brown & Aggleton, 2001; Squire *et al.*, 2007; McTighe *et al.*, 2010), but make different predictions about whether perirhinal cortex (PRh) lesions bias novel objects to appear familiar or, alternatively, bias familiar objects to appear novel. The first part of the present study compared these different predictions.

According to Squire *et al.* (2007), the perirhinal cortex signals object novelty and the hippocampus signals object familiarity. This model, therefore, predicts that PRh lesions will bias novel objects to seem familiar, as judgements are dominated by hippocampal (familiarity) activity. The exploration of novel objects will, therefore, be reduced. A different model (McTighe *et al.*, 2010) assumes that the perirhinal cortex holds object-level representations of complex stimuli. Loss of this area leads to the use of simpler, feature-based representations that are more prone to interference with other stimuli, again resulting in novel objects appearing familiar and so reducing exploration levels for novel objects. A very different possibility is that loss of the perirhinal cortex causes both familiar and novel objects to be perceived as novel, leading to raised levels of exploration (Wiig & Bilkey, 1994). A further possibility (Brown & Aggleton, 2001) is that the perirhinal cortex signals object novelty, with familiarity detection an intrinsic part of this same process, i.e. also signalled by perirhinal cortex. It follows, therefore, that PRh lesions will decrease the exploration of novel objects (novelty detection is compromised) but potentially increase the exploration of familiar objects (as familiarity detection is also compromised). As a result, overall exploration levels may appear unaffected.

To test these different predictions, Experiment 1 examined how rats with PRh lesions behave when presented over successive trials with pairs of objects that are either always novel or always familiar. It was found that PRh lesions had no apparent effects on overall exploration levels when either novel or familiar objects were presented separately (indirect test of recognition), yet impaired one-trial object recognition when novel and familiar objects were simultaneously presented (direct test). This dissociation led to further tests of familiarity learning. The first test was to determine whether the deficit found after PRh lesions on direct measures of object recognition can be overcome when the familiar object is repeatedly presented prior to the test trial with a novel object (Experiment 1c). Additional tests (Experiment 2) then determined the number of object presentations that might be required to compensate for loss of the perirhinal cortex and to test the nature of the information used to guide object recognition in the absence of perirhinal tissue.

## Materials and methods

### Animals

The two series of experiments used 26 male, Lister Hooded rats (Harlan, Bicester, UK), housed in pairs under diurnal conditions (14 h light/10 h dark). Water was provided *ad libitum* throughout the study. Prior to surgery, rats weighed between 282 and 322 g. After a 2-week recovery period, animals were food-deprived to no lower than 85% of their free-feeding body weights and behavioural testing began. All experiments were performed in accordance with the UK Animals (Scientific Procedures) Act, 1986 and associated guidelines.

### Apparatus

**Fig. 1:**
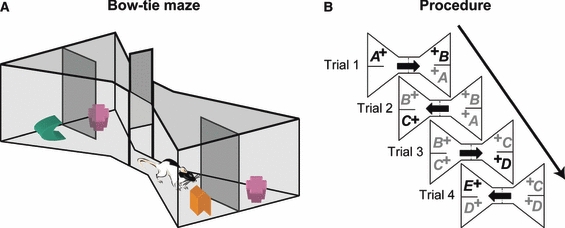
(A) Schematic of the bow-tie maze. A central sliding door separates the two ends of the maze in which two objects are placed. (B) General procedure for the standard object recognition test showing the presentation order of the objects. All objects are rewarded (+). Arrows show direction of rat movements. Bold letters represent novel objects and grey letters represent familiar objects.

For all experiments, rats were tested in a bow-tie-shaped maze made with steel walls and a wooden floor (Fig. 1A). The maze was 120 cm long, 50 cm wide and 50 cm high. Each end of the apparatus was triangular, the apices of which were joined by a narrow corridor (12 cm wide). An opaque sliding door set in the middle of the corridor could be raised by the experimenter. The far wall of each triangle contained two recessed food wells, 3.5 cm in diameter and 2 cm deep. The food wells were separated by a short, opaque dividing wall that protruded 15 cm from the middle of the end wall. These food wells were covered by objects in the experiment proper. Illumination was provided by ceiling lights giving a mean light intensity of 581.0 lx in the centre of the maze.

### Objects

The experiments used numerous junk objects, each differing in shape, texture, size and colour. Every object was large enough to cover a food well but light enough to be displaced. Any object with an obvious scent was excluded. Sufficient objects were used to ensure that no object was repeated across experiments. All objects had multiple, identical copies, so that different copies of the same object were always used when an object was repeated within a session. All objects were cleaned with alcohol wipes after each session.

### Pre-training

All rats were habituated to the maze so that after seven pre-training sessions they would run from one side of the maze to the other and displace an object covering a food well in order to reach food rewards (for fuller description, see Albasser *et al.*, 2010a). Four pairs of objects were used during pre-training, but these objects were not used in any of the following experiments.

### Object recognition – general protocol

Every experimental condition was modified from a standard spontaneous object recognition test in the bow-tie maze (Albasser *et al.*, 2010a). That test is, therefore, described first. Each object recognition session contained multiple trials, and during each trial the animal could freely explore two objects, one novel the other familiar (Fig. 1B). To start each session, a rat was placed on one side of the maze (Trial 0; Table 1A, spontaneous object recognition), where a single object (object A) covered a food well that contained a single sucrose pellet (45 mg; Noyes Purified Rodent Diet, Lancaster, NH, USA). The rat remained in that part of the maze (with object A) for 1 min. The central sliding door was then raised and the rat ran to the opposite side of the maze.

**Table 1:** Sequence of object presentation in Experiment 1. (A) Experiment a (20 trials, two sessions). (B) Experiment b (12 trials, one session) (C) Experiment c (12 trials, three sessions)

(A)													
	Spontaneous object recognition							Novel + Novel object					
		
Trial	0	1	2	3	4	5	6	7	8	9	10	11	12
Objects	**A**	**B**	**C**	**D**	**E**	**F**	**G**	**H**	**J**	**L**	**N**	**P**	**R**
	–	A	B	C	D	E	F	**I**	**K**	**M**	**O**	**Q**	**S**

	Familiar object							Novel vs. Highly familiar					
			
Trial	13	14	15	16	17	18		19	20	21	22	23	24
Objects	**T**	T	T	T	T	T		**U**	**V**	**W**	**X**	**Y**	**Z**
	**T**	T	T	T	T	T		T	T	T	T	T	T

(B)													
	Novel + Novel object						Familiar + Familiar object						
		
Trial	1	2	3	4	5	6	7	8	9	10	11	12	
Objects	**A**	**C**	**E**	**G**	**I**	**K**	**M**	M	M	M	M	M	
	**B**	**D**	**F**	**H**	**J**	**L**	**N**	N	N	N	N	N	

(C)													
		Familiar object						Delay			Novel vs. Highly familiar		
				
Trial		1	2	3	4	5	6	1, 15, or 90 min			7		
Objects		**Z**	Z	Z	Z	Z	Z				**A**		
		**Z**	Z	Z	Z	Z	Z				Z		

Each letter represents a different object, while repeats of the same letter represent identical copies of that object. Within a given trial the novel object is in bold. When an object was repeated, an identical copy was always used.

For standard object recognition trials, for example as part of Experiment 1a, the rat now had a free choice between object A, now familiar, and novel object B (Trial 1; Table 1A; Fig. 1B). Thus, there was a direct comparison between a novel and a familiar object. Each object was concurrently available for the rat to explore for a total of 1 min (Table 1A). The central sliding door was then raised to reveal two more objects (familiar object B vs. novel object C) at the opposite end of the maze for exploration by the rats (Trial 2). After a further 1-min period the door was raised to reveal objects C and D (Trial 3), etc. The retention period between trials was always a maximum of 60 s. Both the familiar and the novel objects always covered a single sucrose pellet, which the rat pushed aside to retrieve. This baiting procedure, which ensured that the objects were approached, did not affect the validity of the recognition test as this relied on the differential exploration of the objects. The placement of objects (including novel objects) varied from left to right according to a pseudorandom schedule. For all experiments, the order of the particular objects used in the test was reversed for half of the rats. This counterbalancing ensured that the novel object in any given pair is reversed; for example for half of the rats in the trial that paired together the following two objects, a toy and a cup, the cup was the novel object. For the remaining rats, the toy was the novel object.

### Experiments 1a–c: object novelty/familiarity tested directly by recognition and tested indirectly by habituation

Experiment 1 consisted of three sub-experiments (Experiments 1a–c). For Experiments 1a and 1b each session was further divided into a number of discrete trial blocks that contained different test conditions.

#### Experiment 1a

Rats received two sessions, each of 24 trials. Each session was divided into four six-trial blocks, each of which involved a different test condition that was repeated over the two sessions (Table 1A), i.e. there were four distinct conditions within each session.

The first condition, ‘standard object recognition’, involved six trials per session. This test of one-trial object recognition was identical to that described in the general protocol (above), i.e. rats were directly tested on their ability to discriminate a novel from a familiar object when both are presented together (Fig. 1; Table 1A, Trials 1–6). The familiar object was presented to the rat on just one previous trial. This procedure has previously been shown to be highly sensitive to PRh lesions (Aggleton *et al.*, 2010; Horne *et al.*, 2010), and so was principally included to confirm the effectiveness of the PRh lesions.

The second condition was ‘novel + novel object exploration’ in which both objects on every trial were novel, i.e. no object was repeated either within or across a trial (Table 1A, Trials 7–12).

In the third condition both objects were familiar and so were repeated across trials. In this ‘familiar object exploration’ condition, the two objects for each trial were identical to each other and copies of this same pair of identical objects were repeated for all six trials, so encouraging habituation of exploration (Table 1A, Trials 13–18).

Finally, in the fourth condition, ‘novel object vs. highly familiar object recognition’, a copy of the repeatedly exposed, highly familiar object from the previous condition (‘familiar object exploration’) was now accompanied on each of six trials by a different novel object (Table 1A, Trials 19–24). Relative exploration of the novel object and the highly familiar object was measured. This final condition assessed how recognition mechanisms might benefit from additional training trials with the familiar object. The left/right position of the familiar object varied across trials. The order in which the blocks of trials were given to the rats was counterbalanced, except that ‘familiar object exploration’ was always followed by the ‘novel object vs. highly familiar object’ condition.

#### Experiment 1b

This experiment involved 12 trials in a single session (Table 1B), divided between two conditions (each six consecutive trials). For both conditions, the trial by trial levels of object exploration were recorded. Six trials repeated the ‘novel + novel object exploration’ condition described above in Experiment 1a. The other six trials constituted the ‘familiar + familiar’ condition, which provided an alternative to the ‘familiar object’ condition of Experiment 1a for examining the habituation of exploration to repeatedly presented objects. Rather than showing the same pair of identical objects trial after trial, as in Experiment 1a (objects T + T, Table 1A), the same pair of different objects was presented on each of six successive trials (e.g. objects M + N, Table 1B). As both objects had to be novel for the first trial of the ‘familiar + familiar’ condition (Trial 7, Table 1B), this particular trial was formally equivalent to Trial 1 of the ‘novel + novel object exploration’ condition. This direct equivalence provides matching baselines from which to compare the impact of object novelty and object repetition. The order in which these two conditions were given to the rats was counterbalanced. Experiment 1b was carried out 3 weeks after completing Experiment 1a.

#### Experiment 1c

Subtests from both Experiments 1a and 1b indicated that rats lacking perirhinal cortex can: (i) habituate to pairs of familiar objects; (ii) show normal levels of exploration of novel objects; and (iii) show effective novelty discrimination (the preferential exploration of novel over familiar objects), but only if given repeated exposures to the familiar object prior to it being paired with a novel object. These findings indicate the existence of non-perirhinal mechanisms that can distinguish novel from familiar object information. The properties of these putative systems are largely unknown. Experiment 1c, therefore, looked at the persistence of this familiarity information with the expectation that it would show a more rapid decrement than perirhinal-based information.

For this reason, the impact of more extended retention delays (15 and 90 min) on object recognition was examined, in addition to the standard < 1-min delay. The rats received three sessions, each of seven trials (Table 1C). The first six trials involved the repeated presentation of the same object (familiar object Z) both within and across trials, i.e. an identical procedure to the ‘familiar object’ condition in Experiment 1a. The sixth trial was followed by a delay of 1, 15 or 90 min. On Trial 7 the rat was presented with a novel object paired against the highly familiar object Z from Trials 1–6. The order of the three delays (1, 15, 90 min) and the identity of the familiar object were counterbalanced between animals (the familiar object was not repeated across sessions). For the retention delays of 15 and 90 min the rat was removed from the apparatus and placed in its home cage, before being re-introduced to the bow-tie maze for Trial 7. There was a 2-week period between Experiment 1c and completing the previous experiment.

### Experiments 2a and b: repeated exposure of the familiar object and its impact on recognition performance by rats with PRh lesions

Experiments investigating the effects of PRh lesions on object recognition have typically involved a single exposure to the familiar object before it is paired with a novel object for the test trial. However, in Experiment 1 we found that discrimination performance (novel vs. familiar) by the lesioned rats was equal to that of the control group when the familiar stimulus had been shown on 12 occasions (six trials) before the test trial. This finding suggests that the disruptive effects of PRh lesions on object recognition may be confined to when the familiar object has only had a limited number of pre-exposure (familiarisation) trials. Determining the required number of familiarisation trials was more systematically tested in Experiment 2.

Experiment 2b also examined whether familiarity learning by rats with PRh lesions is qualitatively different to that in normal rats, i.e. whether the spared recognition processes have different characteristics. One possibility is that rats lacking perirhinal cortex show an abnormal bias to encode novel/familiar object information using non-visual sensory systems (see Winters & Reid, 2010). This particular possibility was tested by examining the rats’ performance when testing was switched to the dark after a series of trials in the light. The prediction was that this sudden demand on somatosensory coding might benefit the animals with PRh lesions if they were biased away from visual cues, leaving them equal or even superior to the control rats when tested in the dark. Experiments 2 started 3 weeks after Experiment 1c, and the time between Experiments 2a and 2b was 2 months.

#### Experiment 2a

Rats received a single session of 20 trials, divided into four blocks of five trials. Each block started with a trial consisting of two dissimilar, novel objects. For the next four trials, one of these objects remained constant while the other object was replaced, i.e. a new, alternative novel object was used on every trial (Table 2A). This arrangement meant that the second trial of each block was formally identical to a standard object recognition trial (e.g. Experiment 1a), but the following trials involved increasing exposure to the familiar object. After each block of five trials, the objects were discarded and new objects used to start the next block of five trials (Table 2A). The identity of the familiar object, i.e. the object repeated for five trials, was counterbalanced, as was the selection of the novel object used on the second trial of each block of five trials. The actual ‘familiar’ object was replaced by identical copies on successive trials, and a new ‘familiar’ object was used for each block of five trials. The left/right position of the familiar object changed across trials.

**Table 2:** Sequence of object presentation in Experiment 2. (A) Experiment a (20 trials, one session). (B) Experiment b (24 trials, one session)

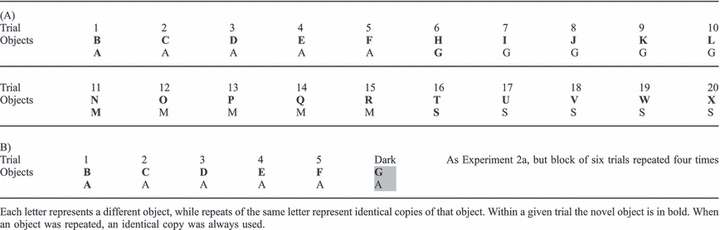

#### Experiment 2b

Rats received a single session of 24 trials, divided into four blocks of six trials. The design of the experiment was identical to Experiment 2a, except that each block of trials had an additional, final test trial in the dark (Table 2B). For these dark trials, all sources of light were switched off and occluded in the test room immediately after Trial 5 of each block, and before the central door was raised. The lights were then turned back on again at the start of the next trial block. During the dark trials the light intensity in the centre of the maze was 0.11 lx. The experimenter wore night-vision goggles (Productive Firm Dipol, Belarus) and the session was recorded with two infra-red cameras (Maplin Electronics, UK) fixed directly above the maze.

### Analysis of behaviour

Animals were video-recorded throughout training. Object exploration was defined as directing the nose at a distance < 1 cm from the object, with the vibrissae moving, and/or touching it with the nose or the paws. Object exploration was not scored when animals sat on the object, when rats used the object to rear upward with the nose of the rat facing the ceiling, or when chewing the object. The duration of exploration was determined by holding down a key pad on a computer during the bursts of exploration recorded on video. For tests of object recognition, two performance indices were calculated, D1 and D2 (Ennaceur & Delacour, 1988). D1 is the duration of exploration time devoted to the novel object minus the exploration time devoted to the familiar object. The second measure (D2) also uses the difference in exploration times (i.e. D1), but then divides D1 by the total duration of exploration given to both the novel and familiar objects. Thereby, the D2 index better compensates for individual changes in amounts of exploration. The resulting D2 ratio can vary between +1 and −1, with a positive ratio showing a preference for novel objects and a ratio of 0 corresponding to no preference, i.e. chance. The ‘updated D2’ was the D2 ratio recalculated after each trial of a block of trials (Experiments 1 and 2). Throughout the experiment the behavioural scoring was blind, i.e. the experimenter did not know the group allocation of the individual rats.

### Surgery

Sixteen rats received bilateral PRh lesions, while 10 rats served as controls. Rats were anaesthetized using an isoflurane–oxygen mixture before being placed in a stereotaxic frame (David Kopf Instruments, Tujunga, CA, USA), with the incisor bar set at 5.0 mm to the horizontal plane. A sagittal incision was made in the scalp, and the skin retracted to expose the skull. The PRh lesions were made by injecting a solution of 0.09 m*N*-methyl-d-aspartate (NMDA; Sigma, Poole, UK) dissolved in phosphate-buffered saline (pH 7.4) in three sites per hemisphere using a 1-μL Hamilton syringe (gauge 26s, outside diameter 0.47 mm) held with a microinjector (Kopf Instruments, Model 5000). Bilateral injections of 0.225 μL NMDA were made at a rate of 0.10 μL/min, with the needle finally left in place for a further 4 min. The coordinates of the injections relative to bregma were: (i) antero-posterior (AP) −1.8, medial-lateral (ML) ± 5.9, dorso-ventral (DV) −9.3; (ii) AP −3.4, ML ± 6.1, DV −9.6; (iii) AP −5.0, ML ± 6.2, DV −9.0. Rats in the surgical control group received identical treatment, except that the dura was repeatedly perforated with a 25-gauge Microlance 3 needle (Becton Dickinson, Drogheda, Ireland) and no fluid was infused into the brain.

### Histology

Following behavioural testing, all rats received a lethal overdose of sodium pentobarbitone (60 mg/kg; Euthatal, Rhone Merieux, UK) and were then transcardially perfused with 0.1 m phosphate-buffered saline (PBS) followed by 4% paraformaldehyde in 0.1 m PBS (PFA). The brains were removed and postfixed in PFA for 4 h, and then transferred to 25% sucrose overnight at room temperature with rotation. Sections were cut at 40 μm on a freezing microtome in the coronal plane and then stained with cresyl violet.

Quantitative estimates were made of the extent of the PRh damage (Table 3). For each animal, images from both hemispheres were taken from 12 coronal sections (i.e. 24 images) along the length of the perirhinal cortex from AP −2.76 to −5.76 (Paxinos & Watson, 2005). These images were captured with a Leica DMRB microscope. Area measurements were made using the program analySIS^D (Soft-Imaging Systems, Olympus). The tissue loss from these 24 images was then summed to produce a total lesion size and then expressed as a percentage of the size of an intact perirhinal cortex (from a control rat). Tissue damage beyond the perirhinal cortex was also measured in area Te2, the piriform cortex and the lateral entorhinal cortex (Table 3).

**Table 3:** The distribution of damage in the 12 rats with bilateral lesions of the perirhinal cortex

Percentage of damage	100–75%	75–50%	50–25%	25–0%
Rostral perirhinal cortex	0	2	**7**	3
Mid perirhinal cortex	**8**	4	0	0
Caudal perirhinal cortex	**10**	2	0	0
Area TEv – at lesion AP	0	0	**6**	**6**
Area TEv – total	0	0	0	**12**
Piriform cortex – at lesion AP	0	0	2	**10**
Piriform cortex – total	0	0	0	**12**
Lateral entorhinal cortex – at lesion AP	3	**5**	4	0
Lateral entorhinal cortex – total	0	0	0	**12**

The percentage of damage was allocated to one of four categories, each of 25%. For each area, the number represents the number of rats falling into one of the four categories. The bold numbers correspond to the modal number for each brain region. The 100–75% category reflects extensive damage, while the 25–0% category reflects considerable sparing. The perirhinal cortex was divided into its rostral, mid and caudal parts. The percentage of extra-perirhinal damage was measured in three adjacent regions using both the size of that region immediately adjacent to the perirhinal cortex (antero-posterior from bregma = −2.76, −6.84) and its overall (total) area (see Materials and methods).

### Statistical analysis

Group comparisons for levels of object recognition (D1 or D2) typically used a *t-*test based on the final cumulative scores (e.g. Experiment 1a). For Experiment 1c, an anova with one between-factor (lesion group) and one within-factor (retention interval) was used to look at forgetting rates. Comparisons of levels of object exploration (Experiments 1 and 2) were also based on a one between (group) by one within (trials) factor anova. When significant interactions were found the simple effects for each condition were analysed as recommended by Winer (1971) using the pooled error term; on occasions when there was a significant main effect but no interaction the simple effects were examined so that regions that significantly differed between groups could be identified (Howell, 1987).

To determine if groups successfully discriminated novel from familiar objects, one sample *t*-tests were conducted using the cumulative recognition indices (D1 and D2) from the completion of test session (e.g. Experiments 1a and 2). These *t*-tests considered if the mean group scores were above chance (zero), i.e. if the group showed a preference for novelty. These one-sample *t*-tests were one-tailed as the only issue was whether scores were above chance. All other *t*-tests (e.g. between lesion groups) were two-tailed.

## Results

The study examined three inter-related questions in the bow-tie maze. (i) Did the PRh lesions cause novel objects to be perceived as familiar? (ii) Did the PRh lesions cause familiar objects to be perceived as novel? (iii) Could the impact of the PRh damage be reversed by additional training? It was also necessary to confirm whether the PRh lesions were sufficient to disrupt standard object recognition.

Experiments 1 and 2 examined all of these issues in an interlinked manner so that the same question was often re-examined in several related conditions. Rather than describe the results in the order that they were conducted, the findings from the 10 different conditions (Tables 1 and 2) have been re-grouped so that all of the relevant findings for each of the above questions are brought together. The concern that this re-grouping might conceal possible order effects can be addressed as: (i) when the same question was tested at different stages of the study the findings were always highly consistent; (ii) wherever possible the order of testing within an experiment was counterbalanced; and (iii) inspection of the data failed to show evidence of order effects. Analyses of all recognition tests looked at both the D1 and D2 indices, but only the D2 results are presented in the main text as all of the significant findings were consistent, irrespective of index. D2 is the preferred index as it compensates for individual differences in total exploration. Comparisons of total exploration times are presented throughout.

### Histology

**Fig. 2:**
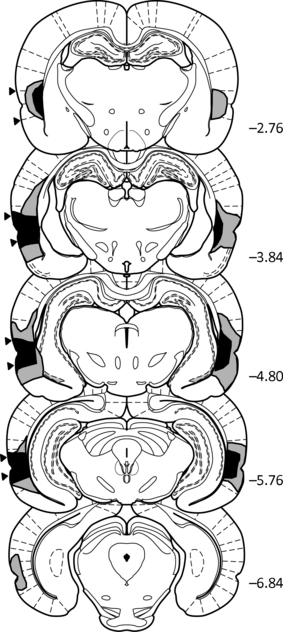
Diagrammatic reconstructions of the PRh lesions showing the individual cases with the largest (grey) and smallest (black) lesions. The numbers refer to the distance (in millimetre) from bregma (adapted from Paxinos & Watson, 2005). The black arrows represent the borders of the perirhinal cortex. The PRh group comprised 12 rats.

Of the 16 original PRh rats, the surgeries in 12 cases removed a considerable extent of perirhinal cortex (Fig. 2). The mean extent of damage using the borders of Burwell (2001) was 65%. Among these 12 cases (Table 3), the only region with sparing in the majority of cases was the rostral border of the perirhinal cortex, a region that is in fact outside the perirhinal cortex as designated by Shi & Cassell (1999). Consequently, the mean extent of damage in the mid and caudal perirhinal cortex, i.e. the perirhinal cortex of Shi & Cassell (1999), was 82%. Table 3 shows that eight of the 12 rats had lost over 75% of the mid perirhinal cortex, with 10 of the 12 rats losing over 75% of the caudal perirhinal cortex (Table 3). The extent of caudal perirhinal cortex damage is significant in view of convergent evidence that this region within areas 35 and 36 is particularly important for object recognition (Albasser *et al.*, 2009, 2010b).

The attempt to make complete PRh lesions inevitably led to some additional damage. In some cases there was involvement of the dorsal and superficial parts of the piriform and lateral entorhinal cortices, often in both hemispheres (Table 3). Ventral area Te2 was sometimes thinned (*n* = 7). In eight rats the lesions extended medially to involve a very restricted portion of caudal CA1, immediately medial to the fundus of the rhinal sulcus (bilateral in two cases). The lesions occasionally extended unilaterally onto more superior cortex (*n* = 4). In five cases there was unilateral damage to rostral postrhinal cortex. The four PRh rats that were excluded had excessive cortical damage (*n* = 3) or only unilateral perirhinal cortex damage (*n* = 1).

### Behavioural findings

#### Do PRh lesions disrupt standard object recognition?

**Fig. 3:**
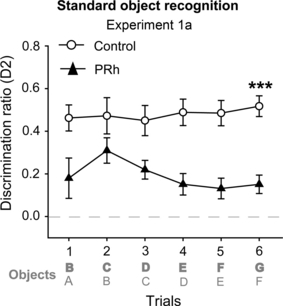
Experiment 1a: standard object recognition by rats with PRh lesions (black triangle) and their controls (white circle). One novel and one familiar object were presented at each trial. The diagram shows the recognition discrimination ratio D2, which was updated after every trial using cumulative data. Scores can range from −1 to +1. Data shown are mean ± standard error of the mean. Group differences ***P< 0.001. The dashed line is chance. In the schematic of the testing protocol (see Table 1) bold letters (upper) represent novel objects and lower letters represent familiar objects.

The first six trials of Experiment 1a examined standard object recognition. The PRh damage produced the expected object recognition impairment (Fig. 3), consistent with many previous studies. Consequently, the final, updated D2 scores of the PRh rats were far lower than those of the control rats (*t*_20_ = 10.65, *P*< 0.001), though the PRh group scores were above chance (one-sample *t*-test, *t*_11_ = 3.48, *P*< 0.005). Total exploration times for the objects used in this condition were comparable between the two groups (*t*_20_ = 1.17, *P*= 0.25).

This one-trial object recognition deficit in the PRh group persisted throughout behavioural testing. In Experiment 2 on the second trial of each block of trials a novel object was paired with an object made familiar from just one previous sample trial (e.g. Trials 2, 7, 13 and 17, Experiment 2a; Table 2). The PRh rats were highly impaired on these specific trials, which taxed one-trial recognition (simple effects second trial: Experiment 2a, *F*_1,80_ = 53.12, *P*< 0.001; Experiment 2b, *F*_1,100_ = 45.52, *P*< 0.001).

### Do PRh lesions cause novel objects to be perceived as familiar?

**Fig. 4:**
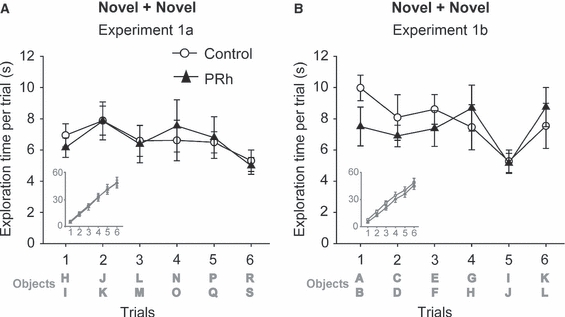
Mean total exploration per trial in the novel + novel condition (two different objects were presented in each trial) in Experiment 1a (A) and Experiment 1b (B). The PRh group is represented by black triangles, the Control group by white circles. The inset graphs in grey show the cumulative exploration (s) across trials. Data shown are mean ± standard error of the mean. The dashed line is chance. In the schematic of the testing protocol (see Table 1) bold letters represent novel objects, i.e. all objects are novel.

The question was whether PRh rats shown novel objects would display rates of exploration lower than those of the controls (as if the objects were perceived as familiar). In Experiment 1a, rats were shown two novel objects on each of Trials 7–12 (‘novel + novel object’; Fig. 4A), but the total amount of exploration (s) devoted to the objects did not differ between the two groups (*F*< 1) or across trials (*F*_5,100_ = 1.64, *P*= 0.16). There was also no group by trial interaction (*F*< 1).

Likewise, Trials 1–6 of Experiment 1b consisted of pairs of novel objects. Once again, overall exploration levels were not affected by the PRh lesions (*F*< 1) or across trials (*F*_5,100_ = 2.12, *P*= 0.09), and no group by interaction was found (*F*_5,100_ = 1.099, *P*= 0.37; Fig. 4B). As novel objects were presented on every trial there was no sign of habituation, i.e. exploration levels did not fall over successive trials.

### Do PRh lesions cause familiar objects to be perceived as novel?

Here, the question was whether rats with PRh lesions show abnormally high levels of exploration for objects that have been previously presented, i.e. behave as though familiar objects are novel. Once again, this question was repeatedly examined in the various conditions presented below. In all cases the result was the same, the PRh rats showed normal levels of exploration when familiar objects are shown in isolation.

**Fig. 5:**
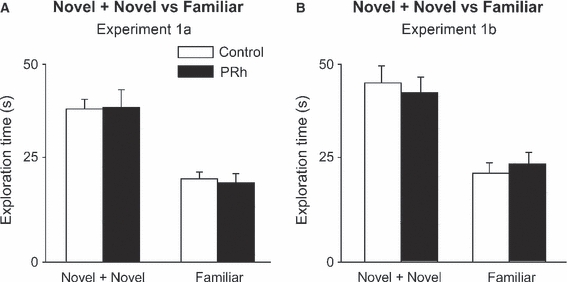
Overall total exploration in seconds of the novel + novel (two different objects were presented in each trial) and the familiar (identical copies of the same objects were presented across trials) conditions in Experiment 1a (A) and Experiment 1b (B). The PRh group is in black and Control group is in white. Data shown are mean ± standard error of the mean.

In Experiment 1a on Trials 13–18 all rats were shown the same two identical objects on every trial (‘familiar object’ condition). Not only were the overall exploration levels of the PRh rats comparable to those of the control rats (*F*< 1; Fig. 5A), but the rats also showed lower overall levels of exploration to these familiar objects than to the novel objects (‘novel + novel object’ condition) in Experiment 1a (*F*_1,20_ = 43.83, *P*< 0.001). Trial by trial analyses for the familiar object condition also failed to find any evidence that the PRh lesions altered patterns of exploration (all group effects *F*< 1).

**Fig. 6:**
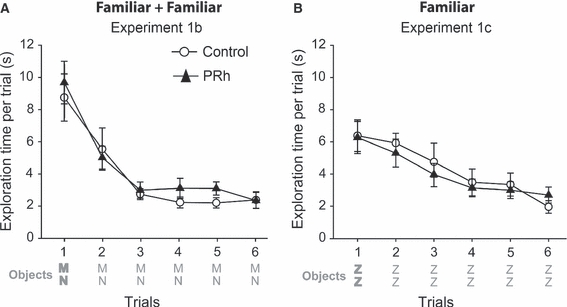
Rates of habituation to repeated objects. Mean total exploration per trial in the familiar + familiar condition (identical copies of two objects were presented in each trial) in Experiment 1b (A) and in the familiar condition (identical copies of only one object were presented across trials) in Experiment 1c (B). The PRh group is represented by black symbols, the Control group by white symbols. Data shown are mean ± standard error of the mean. The dashed line is chance. In the schematic of the testing protocol (see Table 1) bold letters represent novel objects (Trial 1).

The same question was addressed in Experiment 1b but with a small procedural modification (‘familiar + familiar object’). Now, rats were shown two different objects (both initially novel), and these same two objects were then repeatedly shown over the next five trials (Trials 7–12). Once again there was no overall difference between the exploration levels of the two groups (*F*< 1; Fig. 5B), and both groups showed lower overall levels of exploration for the familiar objects than for the ‘novel + novel object’ condition in Trials 1–6 of Experiment 1b (effect of condition, *F*_1,20_ = 63.38, *P*< 0.001). Likewise, there was no evidence of an interaction between lesion and the exploration levels in the two conditions (*F*< 1). That is, the PRh rats showed a seemingly normal discrimination of novel from familiar when the objects were presented sequentially, i.e. tested indirectly. Finally, the levels of exploration of the objects that were repeated (‘familiar + familiar object’) decreased across successive trials (Fig. 6A; trial effect: *F*_5,100_ = 30.36, *P*< 0.001), and this decrease was the same for both groups (Fig. 6A; group effect *F*< 1; group by trial interaction *F*< 1).

The same trial by trial analyses were carried out for the exploration of the ‘familiar object’ in Trials 1–6 of Experiment 1c, except that now this condition was repeated over three separate sessions (with a different object in each session). The data for each individual trial were, therefore, combined. Again, there was no group difference in total levels of object exploration (group effect: *F*< 1). Furthermore, both groups showed a very clear decrease in exploration with repetition of the same object (trial effect: *F*_5,100_ = 31.75, *P*< 0.001; Fig. 6B), and this habituation was not affected by the PRh damage (group by trials interaction: *F*< 1).

### Can the impact of PRh lesions be reversed by additional training?

The testing protocol for the bow-tie maze facilitates the repetition of sample trials prior to an object recognition test as the rat is not handled between trials and the baiting of every object ensures that they are repeatedly visited. The following experiments examined whether sample repetition might reverse the one-trial recognition deficit. While different variants of this manipulation were used in Experiments 1 and 2, the pattern of results was consistent.

#### Experiment 1

**Fig. 7:**
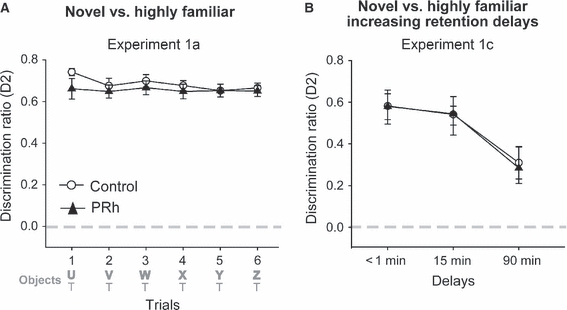
(A) Experiment 1a: discrimination ratio (updated D2) when rats were presented with one novel object and one highly familiar object. (B) Experiment 1c: discrimination ratio (D2) on Trial 7 when rats explored one novel object and one highly familiar object after three retention delays: < 1, 15 or 90 min. The PRh group is represented by black triangles, the Control group by white circles. Scores can range from −1 to +1. Data shown are mean ± standard error of the mean. The dashed line is chance. In the schematic of the testing protocol (see Table 1) bold letters (upper) represent novel objects and lower letters represent familiar objects.

On Trials 19–24 of Experiment 1a every rat was simultaneously presented with a novel object and an object made highly familiar over multiple previous trials (e.g. on Trial 19, object ‘U’ vs. the highly familiar object ‘T’, see Table 1). Throughout this ‘novel object vs. highly familiar object’ condition the rats with PRh lesions displayed a very clear preference for the novel object over the highly familiar object (Fig. 7A). This preference (D2) was comparable in magnitude to that shown by the control rats (*t*< 1). Again, both groups accrued comparable total exploration times with the objects (*t*< 1).

This same condition was repeated in Experiment 1c, i.e. a constant familiar object was presented on Trials 1–6 (Table 1). Now there was a delay of 1, 15 or 90 min before Trial 7, when a novel object was paired with the highly familiar object (‘novel object vs. highly familiar object recognition’). As the retention interval between Trial 6 and Trial 7 increased from 1 to 15 to 90 min there was the expected decrease in Trial 7 D2 scores (*F*_2,40_ = 4.55, *P*= 0.017). There was, however, no group difference (Fig. 7B; *F*< 1). Even after a 90-min interval both the control and PRh groups still had D2 scores that were significantly above chance (one-sample *t*-tests; control rats: *t*_9_ = 3.56, *P*= 0.006; PRh rats: *t*_11_ = 2.59, *P*= 0.025), i.e. both sets of animals could distinguish the novel object. Again, total exploration times did not differ between the two groups (*F*< 1).

#### Experiment 2

In both Experiments 2a and 2b rats were shown a series of novel objects in the light that were paired with a constant alternative object for five trials (e.g. Trials 1–5). After each set of five trials the constant alternative was replaced (e.g. Trials 6–10, see Table 2), giving four blocks, each of five trials in which rats had a simultaneous choice between a novel object and an object made increasingly familiar by being repeated over five trials.

**Fig. 8:**
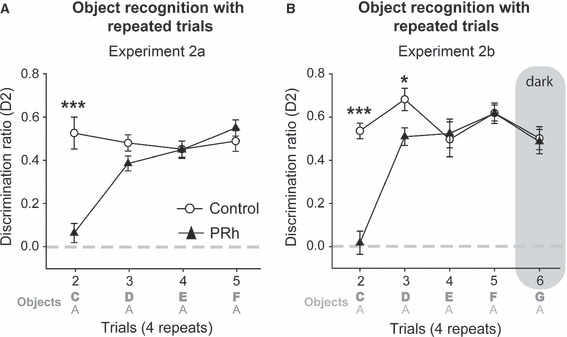
Experiment 2: performance of rats with perirhinal cortex lesions (PRh, black triangles) and their controls (Controls, white circle). (A) The left graph shows the trial by trial discrimination ratio (D2) when a series of novel objects is paired with the same, repeated familiar object for four trials (Experiment 2a). (B) Experiment 2b is a replication of Experiment 2a, except that an additional trial was given with recognition testing for that trial in the dark. Scores can range from −1 to +1. Data shown are mean ± standard error of the mean. Group differences *P< 0.05, ***P< 0.001. The dashed line is chance. In the schematic of the testing protocol (see Table 1) bold letters (upper) represent novel objects and lower letters represent familiar objects.

When all testing was in the light (Experiment 2a), repetition of the familiar object caused the performance of the two groups to converge so that the D2 scores did not differ for Trials 3–5 of each block (simple effects; Trial 3: *F*_1,80_ = 2.24, *P*= 0.14; Trials 4 and 5: *F*< 1), i.e. the marked deficit found for one-trial recognition (see above) disappeared with one additional repetition of the familiar object (Fig. 8A). Comparisons across all four trials showed an overall lesion effect (*F*_1,20_ = 21.22, *P*< 0.001), as well as an effect of trial (*F*_3,60_ = 8.06, *P*< 0.001) and a group by trial interaction (*F*_3,60_ = 12.47, *P*< 0.001) reflecting the group difference confined to Trial 2 (Fig. 8A). Total exploration times across all four trials showed no group difference (*F*< 1), no trial effect (*F*< 1) and no group by trial interaction (*F*< 1).

The final condition (Experiment 2b) was identical to Experiment 2a, but an additional final trial in each block was given in the dark (Table 2B; Fig. 8B). The pattern of discrimination performance for Trials 2–5 was very similar to that seen in Experiment 2a, the only minor difference being that the PRh group were significantly impaired on both Trial 2 (see above) and Trial 3 (group effect, Trials 2–6, i.e. including the dark trial: *F*_1,20_ = 16.55, *P*< 0.001; simple effects, Trial 3: *F*_1,100_ = 5.05, *P*= 0.027), i.e. it took an extra familiarisation trial for the PRh rats to reach the D2 scores of the control rats (Fig. 8B). This deficit was limited to Trials 2 and 3, and so explained the group by trial interaction (*F*_4,80_ = 8.65, *P*< 0.001). Again, total exploration times across all five trials (2–6) showed no group difference (*F*_1,20_ = 1.11, *P*= 0.31).

Switching the last trial into the dark (Fig. 8B) produced a small, but significant, fall in the discrimination index D2, as shown by the comparisons between Trial 5 (light) and Trial 6 (dark; *F*_1,20_ = 5.54, *P*= 0.029). This change was accompanied by an increase in overall exploration time in the dark. There was, however, no lesion effect (*F*< 1) and no group by trial interaction (*F*< 1).

### Lesion extent and performance (Experiments 1 and 2)

Despite their consistent deficit on one-trial object recognition (‘standard object recognition’), the PRh group appeared to perform normally across an array of other conditions. Inspection of the data from those later conditions where there was sparing of performance, for example Experiment 1c (Fig. 7), and Trial 3 of Experiments 2a and Trial 4 of Experiment 2b (Fig. 8) showed that these null results were not due to a subgroup of rats with excessive perirhinal sparing. This conclusion is supported by the fact that the performances of the lesion and control groups on the latter trials of Experiment 2 were seemingly indistinguishable (Fig. 8A and B). Lastly, there was no evidence that performance in Experiments 1 or 2 correlated with the degree of sparing in the rostral perirhinal cortex (the only region to show any consistent sparing). Likewise, there was no significant correlation between performance and the overall perirhinal cortex damage or with the overall perirhinal plus entorhinal cortex damage.

## Discussion

The first goal was to determine whether rats with PRh lesions perceive novel objects as familiar (Squire *et al.*, 2007; McTighe *et al.*, 2010) or perceive familiar objects as novel (Wiig & Bilkey, 1994). Neither outcome occurred as rats with PRh lesions showed normal exploration levels to both novel and familiar objects when presented separately. These findings also appear inconsistent with the prediction that PRh lesions will alter exploration levels for both novel and familiar objects (Brown & Aggleton, 2001).

These null results were not due to the insensitivity of the behavioural measures. Rats displayed clear decreases in exploration with object repetition, which contrasted with higher exploration levels when shown just novel objects (Fig. 5). Furthermore, the PRh lesions induced severe object recognition deficits whenever a direct choice was given between a novel object and an object made familiar by a single previous exposure trial (Experiments 1a, 2a and 2b). This one-trial object recognition memory deficit is characteristic of PRh lesions (Mumby & Pinel, 1994; Ennaceur *et al.*, 1996; Aggleton *et al.*, 1997, 2010; Norman & Eacott, 2004; Barker *et al.*, 2007; Winters *et al.*, 2008).

A potential concern is that the normal, overall exploration levels of the PRh rats reflect special features of the bow-tie protocol. This concern can be tested by considering the exploration levels of rats with PRh lesions from other studies. In all spontaneous object recognition tests there is initially a familiarisation (‘sample’ or ‘study’) phase where rats are standardly given two copies of the same novel object (e.g. A + A). Typically, PRh lesions do not alter rates of sample exploration (Ennaceur *et al.*, 1996; Aggleton *et al.*, 1997; Moran & Dalrymple-Alford, 2003; Winters *et al.*, 2004; Bartko *et al.*, 2007a,b; Mumby *et al.*, 2007; Albasser *et al.*, 2009; McTighe *et al.*, 2010), i.e. these findings accord with those of the present study.

Other support comes from the test phase of spontaneous object recognition tasks, where there is one novel object (B) and one familiar object (A). Once again, overall exploration levels by rats with PRh lesions typically appear normal (Ennaceur *et al.*, 1996; Moran & Dalrymple-Alford, 2003; Aggleton *et al.*, 2010; Horne *et al.*, 2010), despite reduced preference for novel object B over familiar object A (see also Experiment 1a, ‘object recognition’). For the latter result to occur, PRh lesions must increase exploration of the familiar object while decreasing exploration of the novel object. Finally, a small number of studies have reported exploration or orientation levels when rats are repeatedly exposed to the same object (Mumby *et al.*, 2007; Albasser *et al.*, 2009) or the same sensory stimulus (Bucci & Burwell, 2004; Robinson *et al.*, 2009) when it is not selectively associated with a reward outcome. Again, as in the present study, there was no PRh lesions effect.

The current findings do, however, conflict with a recent report that rats with PRh lesions given just novel objects in the test phase show reduced exploration, i.e. that novel objects are perceived as familiar (McTighe *et al.*, 2010). This discrepancy between studies requires explanation. It is, therefore, notable that both studies used the same strain of rats, and that the surgical procedures and resultant lesions appear to be very similar. There are, however, a number of procedural differences. Unlike the present study, none of the objects was associated with a food reward (McTighe *et al.*, 2010). At first sight this may seem a critical difference, but it must be remembered that the rats in the present study showed very reliable decreases in object exploration with object repetition, i.e. merely placing a food reward under an object was not sufficient to sustain exploration. Also, as noted above, seemingly normal rates of habituation to objects have been seen after PRh lesions when no rewards were available (Mumby *et al.*, 2007; Albasser *et al.*, 2009). A second difference was that the present testing procedure was continuous, so that the rats were not handled between trials and testing occurred within the same session. Advantages with this method include the increase in trial numbers, so individual object effects can be minimised, and the reduction in any unwanted distraction between trials. In contrast, McTighe *et al.* (2010) employed discrete study and test phases, separated by removal of the rat into a holding cage for 1 h. While this longer retention period might be important, other studies involving habituation to objects after lengthy intervals do not seem to support this view (Mumby *et al.*, 2007; Albasser *et al.*, 2009).

A further consequence of the continuous testing procedure in the present study was that the absolute position of the same object often switched from one end of the maze to the other. This situation did not arise in the study by McTighe *et al.* (2010), where rats always started from the stem of a Y-shaped maze. The potential impact of this spatial variable in the present study was probably reduced by the high opaque walls of the bow-tie maze and the use of multiple trials within each session. Indeed, repeated pilot studies have failed to find evidence that rats can link specific objects with their location using this protocol, a result consistent with the finding that hippocampal lesions do not affect object recognition in the bow-tie maze, despite the familiar objects being placed in new locations (Albasser *et al.*, 2010a).

A potentially telling point is that although the PRh rats in the study by McTighe *et al.* (2010) showed reduced exploration levels in the test phase with both familiar and novel objects (when the delay period was in the light), the same rats showed normal levels of exploration to novel objects in the sample (‘study’) phase, i.e. when first exposed to these objects. As noted above, this finding not only accords with the results of the present study but with almost all of those previous studies of PRh lesions where the sample data are presented (Ennaceur *et al.*, 1996; Aggleton *et al.*, 1997; Moran & Dalrymple-Alford, 2003; Winters *et al.*, 2004; Bartko *et al.*, 2007a,b; Mumby *et al.*, 2007; Albasser *et al.*, 2009; McTighe *et al.*, 2010). Crucially, the data from these studies seem to contradict the conclusion that novel objects are treated as familiar after PRh lesions (McTighe *et al.*, 2010), as exploration in the study phase should surely have been reduced. Instead, the present study and those studies where the data are available repeatedly show that PRh damage do not typically affect overall levels of exploration for either novel or familiar objects. The next step is to examine more formally some of the procedural differences between the present task and that used by McTighe *et al.* (2010), and so better understand factors that promote confusion between novel and familiar objects.

The second goal concerned the impact of multiple familiarisation trials. Amnesics can often overcome their recognition deficit when given extended or repeated opportunities to learn the target stimuli (Huppert & Piercy, 1978; Mayes *et al.*, 1988). Examples include HM who, after extended sample periods, showed normal levels of forced-choice recognition after a retention delay as long as 1 week (Freed *et al.*, 1987). An analogous effect was examined in the present study where, after extra sample trials, rats with PRh lesions could now discriminate between simultaneously presented novel and familiar objects at seemingly normal levels after delays of up to 90 min (Experiment 1c). Experiment 2 then showed that these rats required only one, or at most two, extra familiarisation trials to reach control levels on this measure, and that the available recognition information included non-visual cues as the animals could transfer from a sample in the light to a recognition test in the dark. It is tempting to conclude that these results show that familiarity information is intact after PRh lesions, i.e. novelty is selectively lost, so that the rats show normal declines with object repetition and successful recognition when finally challenged with a novel object. This account fails, however, to explain the normal levels of exploration that the PRh rats gave to just novel objects.

Other evidence that repeated exposures can aid rats with PRh lesions comes from a study where rats received five familiarisation sessions of 5 min each (Mumby *et al.*, 2007). The lesioned rats successful recognised the familiar objects after a 24-h delay, though a deficit was seen after a 3-week retention period (Mumby *et al.*, 2007). Together, these findings highlight subsidiary mechanisms for recognition. One source could be spared perirhinal tissue, but this seems unlikely given: (i) the completeness of the lesions in the mid and caudal perirhinal cortex (see Albasser *et al.*, 2009, 2010b); and (ii) the finding that the rats with the largest PRh lesions still showed effective recognition after object repetition, e.g. Experiment 2. Other structures that could potentially support recognition include the hippocampus (Squire *et al.*, 2007), the medial diencephalon (Aggleton & Brown, 1999), area Te (Miyashita & Chang, 1988; Zhu *et al.*, 1995; Wan *et al.*, 1999), other parts of the ventral visual stream (Bussey *et al.*, 2005) and the parietal cortex (Winters & Reid, 2010). Thus, while perirhinal cortex appears pre-eminent for visual recognition, these other regions may require only one or two additional sample trials to become effective.

From this conclusion, it might be supposed that extending the duration of a single sample trial will largely eliminate PRh lesion effects in rats on object recognition. In fact, while normal rats show a positive relationship between extent of exploration in a single trial and subsequent levels of object recognition, this correlation disappears after PRh lesions (Albasser *et al.*, 2009). This result suggests that the critical factor is not the length of the trial but the number of sample trials. This preliminary conclusion agrees with the fact that the exploration periods in the present experiment (1 min) are shorter than those in standard object recognition tasks (e.g. Ennaceur *et al.*, 1996; Aggleton *et al.*, 1997; Norman & Eacott, 2004; Barker *et al.*, 2007), yet PRh lesions deficits are still present when using these standard procedures with longer sample periods, i.e. if a small increase in exploration times counteracts PRh lesions effects then this sparing action would be observed in those experiments employing longer familiarisation trials (e.g. 3–5 min against the 1 min of the present study).

The effectiveness of these subsidiary recognition mechanisms may explain the apparent normal sensitivity of the rats to novelty and familiarity when measured indirectly. The assumption is that non-perirhinal regions can set appropriate levels of exploration to novel or familiar objects but do not initially guide preferential choice behaviour between these objects. The underlying implication, that habituation and recognition can be dissociated after PRh lesions (Robinson *et al.*, 2009), is consistent with taxonomies of human memory (Squire & Zola-Morgan, 1991; Squire *et al.*, 2007) that separate recognition memory (declarative) from habituation (non-declarative). This distinction need not, however, be complete if the same subsidiary mechanisms that guide levels of exploration to individual novel and individual familiar objects might aid recognition judgements. This conclusion agrees with growing evidence that implicit, i.e. non-declarative processes, can sometimes contribute to human recognition (e.g. Voss & Paller, 2009). This potential overlap of processes may prove particularly problematic for interpreting spontaneous object recognition tests where both recognition and habituation are measured by the same behavioural index – changes in exploration. These findings reinforce the additional value of choice measures, e.g. by delayed non-matching-to-sample (Steckler *et al.*, 1998), to study rodent recognition memory. At the same time, the present findings highlight shortcomings with current models of perirhinal function as they fail to predict the unaffected exploration levels on all indirect tests of recognition.

## Abbreviations

NMDA: *N*-methyl-d-aspartate
PBS: phosphate-buffered saline
PFA: paraformaldehyde
PRh: perirhinal cortex
